# Sex and Age Differences Modulate Association of Vitamin D with Serum Triglyceride Levels

**DOI:** 10.3390/jpm12030440

**Published:** 2022-03-11

**Authors:** Ying-Lien Cheng, Ting-Wei Lee, Ting-I Lee, Yu-Hsun Kao, Chih-Yin Wu, Yi-Jen Chen

**Affiliations:** 1 Division of Endocrinology and Metabolism, Department of Internal Medicine, Wan Fang Hospital, Taipei Medical University, Taipei 11696, Taiwan; elaine050033@yahoo.com.tw (Y.-L.C.); b8801138@tmu.edu.tw (T.-W.L.); agleems29@gmail.com (T.-I.L.); 2 Division of Endocrinology and Metabolism, Department of Internal Medicine, School of Medicine, College of Medicine, Taipei Medical University, Taipei 11031, Taiwan; 3 Department of General Medicine, School of Medicine, College of Medicine, Taipei Medical University, Taipei 11031, Taiwan; 4 Graduate Institute of Clinical Medicine, College of Medicine, Taipei Medical University, Taipei 11031, Taiwan; 5 Department of Medical Education and Research, Wan Fang Hospital, Taipei Medical University, Taipei 11696, Taiwan; 6 Department of Family Medicine, Wan Fang Hospital, Taipei Medical University, Taipei 11696, Taiwan; gigi710122@gmail.com; 7 Cardiovascular Research Center, Wan Fang Hospital, Taipei Medical University, Taipei 11696, Taiwan; 8 Taipei Heart Institute, Taipei Medical University, Taipei 11031, Taiwan

**Keywords:** age, sex, lipid, risk factors, triglyceride, vitamin D deficiency

## Abstract

The sex and age differences in the relationship between vitamin D and lipid levels remain unclear. This retrospective study investigated the correlations between serum 25-hydroxyvitamin D levels and various biomarkers, along with the sex and age differences in these associations, among 573 men and 436 women during physical check-ups. The mean age of the study population was 51.4 years, and 66% of people had serum 25(OH)D levels below 30 ng/mL. People aged over 65 years had higher 25(OH)D levels than those younger than 65 years, and women had lower 25(OH)D levels than men. Younger age (odds ratio (OR) per year = 1.044, 95% CI, 1.029–1.059, *p* < 0.0001), female sex (OR = 1.779, 95% CI, 1.149–2.755, *p* = 0.0097), and elevated serum triglyceride (TG) levels (OR per 1 mg/dL = 1.005, 95% CI, 1.002–1.007, *p* = 0.0002) were all independent risk factors for vitamin D deficiency. Serum 25(OH)D levels were inversely associated with TG levels. The positive association between vitamin D deficiency and hypertriglyceridemia was significant in men (not in women) and in those aged between 50 and 65 years. In conclusion, younger individuals, women, and middle-aged men with hypertriglyceridemia are at higher risk of vitamin D deficiency.

## 1. Introduction

Vitamin D deficiency is a common disorder. The prevalence of vitamin D deficiency, defined as serum 25-hydroxyvitamin D (25(OH)D) levels <30 ng/mL, is estimated to be 30–80% among adults worldwide [1,2]. Vitamin D not only regulates bone and mineral metabolism, but also exerts various biological effects, including antioxidative, anti-inflammatory, antimicrobial, lipid-lowering, and cardiovascular protective effects [3,4,5]. In addition to osteoporosis, vitamin D deficiency has been reported to be associated with hypertension, diabetes mellitus, dyslipidemia, obesity, and metabolic syndrome [6,7,8]. Women and older adults are particularly vulnerable to vitamin D deficiency [9]. East Asian women commonly avoid sunlight exposure to obtain fair skin, putting them at a higher risk of vitamin D deficiency than men [10,11,12]. Malabsorption of cholecalciferol and ergocalciferol, a decrease in the cutaneous synthesis of vitamin D, reduced exposure to sunlight and an increase in the prevalence of liver and kidney disease all cause serum 25(OH)D levels to decline with age [13,14].

Humans obtain vitamin D through sunlight exposure and food intake. Vitamin D from cutaneous synthesis, or from the diet, is biologically inert. It is converted to 25(OH)D by 25-hydroxylase in the liver. Moreover, 25(OH)D, which is measured to determine an individual’s vitamin D status, requires further hydroxylation in the kidneys to become bioactive vitamin D metabolite [4]. Generally, ultraviolet B irradiation of 7-dehydrocholesterol in the skin contributes to the major source of vitamin D in humans since only a few foods, such as oily fish, egg yolks, and mushrooms, contain significant amounts of vitamin D [15,16]. Therefore, variations in daylight throughout the year and latitude of residence both affect solar radiation, thus influencing vitamin D synthesis. A study involving 95,137 subjects in Korea revealed that the pattern of seasonal fluctuations of serum 25(OH)D levels and solar insolation was similar, suggesting that solar radiation is closely related to serum 25(OH)D levels [17]. The serum 25(OH)D levels are highest during late summer and lowest during late winter in northern countries [18,19,20]. Previous investigations have shown that the serum 25(OH)D levels in winter may be decreased by approximately 25–50% on average as compared to those in summer [21,22]. Accordingly, seasonal variations should be taken into consideration when measuring serum 25(OH)D levels and treating vitamin D deficiency.

Although vitamin D deficiency is common and linked to many chronic disorders, evidence to recommend the routine screening of individuals for vitamin D deficiency is currently insufficient. Therefore, biomarkers to identify individuals at a high risk for vitamin D deficiency could be clinically useful. Characteristic values of such biomarkers could increase a clinician’s suspicion of the presence of vitamin D deficiency as well as its clinical consequences or related comorbidities. Biochemistry is simple and widely used for assessing a patient’s general condition, and the conventional biochemical parameters may include a biomarker of vitamin D deficiency. This study investigated the associations between serum vitamin D levels and various biomarkers, as well as the sex and age differences in these associations.

## 2. Methods

### 2.1. Participants

From March 2018 to February 2020, we collected blood samples from individuals aged > 18 years who had received blood tests for vitamin D during their physical check-ups. Individuals with incomplete laboratory data were excluded. A total of 1009 participants were included in this study. The mean age was 51.4 years, and 56.7% of the participants were male. They were divided into vitamin D sufficient and deficient groups at a cutoff 25(OH)D value of 30 ng/mL. According to the meteorological data of Taiwan, the solar irradiation was relatively lower from November to April (defined as extended winter) and higher from May to October (defined as extended summer) in Taipei [23]. This study was approved by the Taipei Medical University Joint Institutional Review Board (protocol code: N202006010).

### 2.2. Biochemical Measurements

The following data were recorded: age, sex, body weight, body mass index (BMI), complete blood count (CBC), serum levels of fasting plasma glucose, glycated hemoglobin (HbA1c), blood urea nitrogen (BUN), creatinine, uric acid, aspartate transaminase (AST), alanine transaminase, γ-glutamyl transpeptidase, alkaline phosphatase (Alk-p), total bilirubin, total protein, albumin, globulin, triglycerides (TG), total cholesterol (TC), high-density lipoprotein cholesterol (HDL-C), low-density lipoprotein cholesterol (LDL-C), thyroid-stimulating hormone (TSH), triiodothyronine, free thyroxine, and 25(OH)D.

### 2.3. Statistical Analysis

Continuous variables with normal distributions are expressed as means and standard deviations, whereas categorical variables are expressed as frequencies and percentages. The *t* test (normally distributed variables) and chi-square test (categorical variables) were used to analyze the differences between groups. Logistic regression analysis was performed to evaluate the independent biochemical parameters associated with serum 25(OH)D levels, and the data are expressed as odds ratios (ORs). Additionally, linear regression analysis was used to study the association between independent biochemical parameters and serum 25(OH)D levels. All statistical analyses were performed using SPSS version 22.0 (SPSS Inc., Chicago, IL, USA). A *p* value of <0.05 was considered to be statistically significant.

## 3. Results

### 3.1. General Characteristics

The prevalence of vitamin D deficiency (25(OH)D < 30 ng/mL) was 66% among 1009 participants. In our sample, 527 participants had their serum 25(OH)D measurement during extended summer, and 482 participants had measurements during extended winter. The mean serum 25(OH)D levels during extended summer were considerably higher than those during extended winter (37.38 ng/mL vs. 31.33 ng/mL, *p* < 0.05). However, the proportion of 25(OH)D measurements conducted during extended summer was significantly higher in the vitamin D sufficient group than that in the vitamin D deficient group (56.7% vs. 49.9%, *p* < 0.05). As compared to individuals with serum 25(OH)D ≥ 30 ng/mL, those with vitamin D deficiency were younger and constituted a higher percentage of females (Table 1). The CBC data indicated that participants with vitamin D deficiency had significantly lower serum hemoglobin (Hb), hematocrit (Hct), mean corpuscular Hb (MCH), and mean corpuscular volume (MCV) but higher serum platelet levels than those without the deficiency. Moreover, biochemical analyses revealed that patients with vitamin D deficiency had lower HbA1c, BUN, creatinine, and AST levels but higher serum total protein, TG, and TSH levels than those of people with sufficient vitamin D. However, body weight, BMI, and serum levels of fasting plasma glucose, TC, HDL-C, and LDL-C were similar between the vitamin D deficient and sufficient groups.

### 3.2. Sex Differences Associated with Serum Vitamin D

As summarized in Table 2, we investigated the sex differences in the associations between vitamin D deficiency and biomarkers and found that both men and women with vitamin D deficiency had lower HDL-C levels and higher body weights, BMIs, platelet counts, and TG levels than those with sufficient vitamin D. Only men with vitamin D deficiency had higher serum white blood cell (WBC) counts and Alk-p levels than their counterparts with sufficient vitamin D. Moreover, vitamin D deficiency was significantly associated with lower MCV and serum MCH, Hb, Hct, BUN, AST, and TC levels in women.

### 3.3. Age Associated with Serum Vitamin D

We analyzed the association of age as part of the relationship between vitamin D deficiency and biomarkers. In adults aged over 65 years, vitamin D deficiency was associated with significantly higher BMI and WBC levels than sufficient vitamin D (Table 3). By contrast, among individuals younger than 65 years, vitamin D deficiency was associated with lower MCV and serum MCH, Hb, Hct, BUN, creatinine, and AST levels and higher serum platelet counts and total protein, globulin, TG, and TSH levels than vitamin D sufficiency.

### 3.4. Biomarkers Independently Associated with Serum Vitamin D: Age, Sex, and Triglycerides

**Figure 1:**
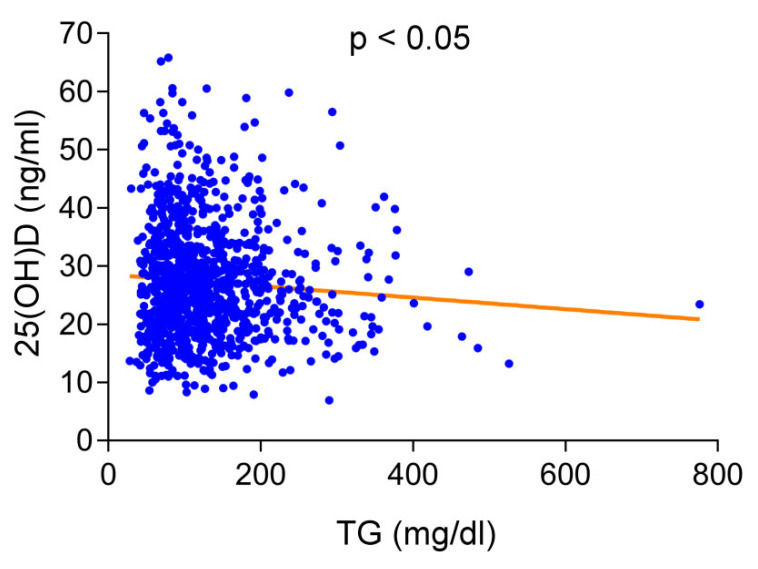
Correlations between serum levels of 25-hydroxyvitamin D (25(OH)D) and triglyceride (TG) levels in total study population. Increased serum TG levels were significantly associated with decreased 25(OH)D levels.

We performed logistic regression analysis and found that only younger age (OR per year = 1.004, 95% CI, 1.029–1.059, *p* < 0.001), female sex (OR = 1.779, 95% CI, 1.149–2.755, *p* < 0.01), and elevated serum TG levels (1 mg/dL, OR = 1.005, 95% CI, 1.002–1.007, *p* < 0.001) were independent risk factors for vitamin D deficiency. The serum vitamin D level of people over 65 years old was significantly higher than that of people under 65 years old (32.3 ± 10 vs. 26.3 ± 9.4 ng/mL, *p* < 0.001). Women had lower vitamin D levels than men (25.0 ± 9.4 vs. 29.0 ± 9.8 ng/mL, *p* < 0.001). As displayed in Figure 1, elevated serum TG levels were significantly associated with decreased 25(OH)D levels (*p* < 0.05). The prevalence of vitamin D deficiency in people with TG levels > 200 mg/dL was significantly higher than that of people with TG levels < 200 mg/dL (74.6% vs. 64.6%, *p* < 0.05).

### 3.5. Relationship between Vitamin D and TG Levels Differs by Sex and Age

**Figure 2:**
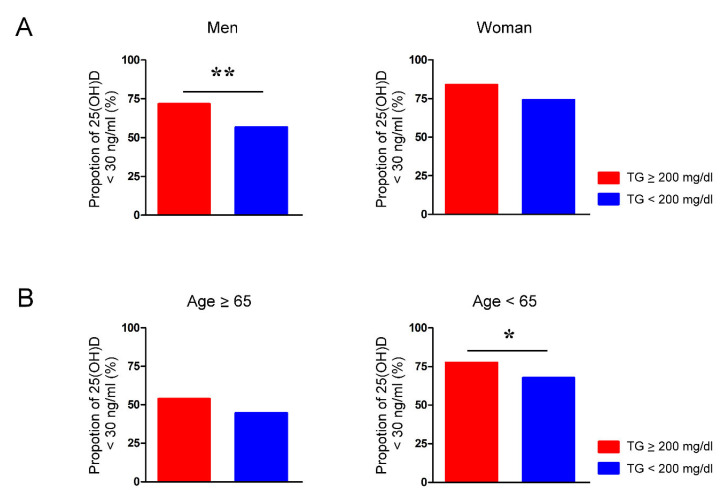
Proportion of vitamin D deficiency in people with and without hypertriglyceridemia is modulated by sex and age. The rate of serum 25-hydroxyvitamin D (25(OH)D) levels below 30 ng/mL in (**A**) men and women and (**B**) individuals aged younger and older than 65 years whose serum levels of triglycerides (TG) were greater or less than 200 mg/dL. * *p* < 0.05, ** *p* < 0.01.

The vitamin D deficiency rate of men with TG levels >200 mg/dL was significantly higher than that of men with TG levels < 200 mg/dL (71.6% vs. 56.7%, *p* < 0.01) (Figure 2A). Women with TG levels > 200 mg/dL and women with TG levels < 200 mg/dL had similar rates of vitamin D deficiency (83.9% vs. 74.1%, *p* = 0.22). We also found that the vitamin D deficiency rate of adults under 65 years of age was significantly higher when the TG levels were >200 mg/dL than when the TG levels were < 200 mg/dL (77.5% vs. 67.6%, *p* < 0.05) (Figure 2B). In contrast, the prevalence of vitamin D deficiency in people over 65 years of age with TG levels >200 mg/dL and TG levels < 200 mg/dL was similar (53.8% vs. 44.5%, *p* = 0.52). 

Additionally, we analyzed the risk of hypertriglyceridemia for vitamin D deficiency in subgroups; we found greater risks for vitamin D deficiency in men and in people aged younger than 65 years with elevated serum TG levels (male sex, 1 mg/dL, OR = 1.004, 95% CI, 1.002–1.007, *p* < 0.001; female sex, 1 mg/dL, OR = 1.004, 95% CI, 1.000–1.009, *p* = 0.049; age < 65 years, 1 mg/dL, OR = 1.002, 95% CI, 1.000–1.005, *p* = 0.029; age ≥ 65 years, 1 mg/dL, OR = 1.005, 95% CI, 0.999–1.010, *p* = 0.099).

### 3.6. Vitamin D Deficiency Is Associated with Hypertriglyceridemia Only among Middle-Aged Men

**Figure 3:**
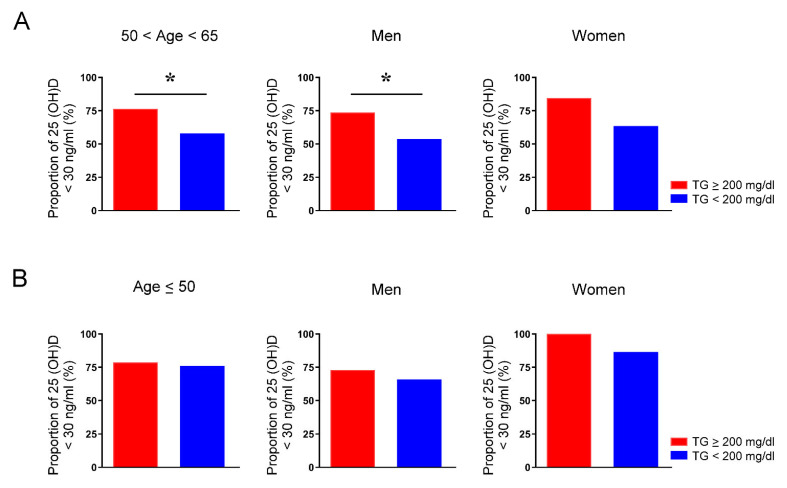
Proportion of vitamin D deficiency in people aged between 50 and 65 years or younger than 50 years with and without hypertriglyceridemia. The rate of serum 25-hydroxyvitamin D (25(OH)D) levels below 30 ng/mL of individuals in total, men, and women aged between 50 and 65 years (**A**) and younger than 50 years (**B**) whose serum levels of triglycerides (TG) were greater or less than 200 mg/dL. * *p* < 0.05.

Because our results indicated that younger people are at higher risk of vitamin D deficiency, we analyzed the sex differences in the associations between vitamin D deficiency and biomarkers in participants younger than 50 years (mean age: 41.7 years). We found that only women with vitamin D deficiency had lower serum MCH, MCV, and creatinine but higher Alk-p levels than their counterparts with sufficient vitamin D (Table 4). Both men and women with vitamin D deficiency had lower HDL-C levels. However, serum levels of TG were similar between the vitamin D deficient and sufficient groups in men and in women. Our findings suggest that vitamin D deficiency is associated with hypertriglyceridemia only among middle-aged people. Additionally, we investigated participants aged between 50 and 65 years and found that men with vitamin D deficiency had significantly higher serum TG levels than those with vitamin D sufficiency (Table 5). By contrast, women with and without vitamin D deficiency had similar TG levels. Moreover, the vitamin D deficiency rate of people aged 50–65 years was significantly higher when the TG levels were >200 mg/dL than when the TG levels were < 200 mg/dL (76.4% vs. 58.1%, *p* < 0.05) (Figure 3A). The vitamin D deficiency rate of men aged 50–65 years with TG levels > 200 mg/dL was significantly higher than that of men with TG levels < 200 mg/dL (73.8% vs. 53.9%, *p* < 0.05). Women aged 50–65 years with TG levels greater or less than 200 mg/dL had similar rates of vitamin D deficiency (84.6% vs. 63.5%, *p* = 0.22). In people younger than 50 years, the vitamin D deficiency rate was similar between those with TG levels > 200 mg/dL and TG levels < 200 mg/dL (78.7% vs. 76.1%, *p* = 0.74) (Figure 3B). The proportion of vitamin D deficiency in adults younger than 50 years with and without hypertriglyceridemia was similar in men (72.9% vs. 65.8%, *p* = 0.40) and in women (100% vs. 86.5%, *p* = 0.38).

## 4. Discussion

An analysis of the National Health and Nutrition Examination Survey in the United States revealed that 14.8% of people aged older than 65 years had vitamin D deficiency and 39.9% of participants aged ≥ 20 years had vitamin D deficiency [7]. The Korea National Health and Nutrition Examination Survey revealed that vitamin D insufficiency was most prevalent between the ages of 20 and 29 years, with a prevalence of 65.0% in men and 79.9% in women [24]. Similarly, we found that people younger than 65 years of age was associated with lower levels of vitamin D, contradicting the generally accepted notion that older adults tend to have more vitamin D deficiency. One reason that older adults may be less likely to have vitamin D deficiency is that they generally have greater health awareness and may eat healthier diets that contain more vitamin D. In addition, they may consume nutrient supplements that contain vitamin D; this result may be supported by the higher vitamin B12 levels observed in older adults with vitamin D sufficiency (data not shown). Moreover, young urban people must often spend considerable amounts of time indoors for work and may engage in few outdoor activities, leading to low sun exposure and a consequently higher risk of vitamin D deficiency. Our study showed that participants had higher serum 25(OH)D levels during extended summer than those during extended winter, which was similar to previous findings reporting the seasonal variations in serum 25(OH)D concentrations [19,21,22]. In the present study, we also observed that women are more prone to vitamin D deficiency than men; this result is consistent with several other studies [25,26,27]. The higher prevalence of vitamin D deficiency among women that we discovered may be attributable to women receiving less sun exposure because light skin is often associated with beauty in Eastern cultures. 

Several clinical studies have indicated the associations among vitamin D deficiency, anemia, and higher platelet counts [28,29,30]. Vitamin D may improve anemia through downregulating hepcidin and promoting erythropoiesis [31]. The anti-inflammatory, anti-oxidant, and anti-thrombogenic potentials of vitamin D are also involved in possible mechanisms for the inverse correlation of platelet counts with serum vitamin D levels [28]. In this study, we found that participants with vitamin D deficiency had significantly lower serum Hb, Hct, MCH, and MCV. However, this association was primarily seen in women, but not in men. By contrast, our study demonstrated that participants with vitamin D deficiency had higher platelet counts, regardless of sex. Furthermore, the associations between vitamin D deficiency and anemia or higher platelet counts were both significant in those who were younger than 65, but not in those older than 65 years. Our findings suggest that vitamin D deficiency may be included in the differential diagnosis of anemia and thrombocytosis, especially in women or in subjects aged younger than 65 years.

Vitamin D deficiency has been linked to dyslipidemia [32,33,34]. Serum 25(OH)D levels were negatively correlated with TG and LDL-C levels, but positively correlated with HDL-C levels [32]. A meta-analysis of 41 randomized controlled trials with a total of 3434 participants revealed that vitamin D supplementation appeared to reduce serum TC, LDL-C, and TG levels [35]. However, whether sex or age affects the association of vitamin D deficiency with dyslipidemia remains unclear. In this study, we found that individuals with serum 25(OH)D < 30 ng/mL had significantly higher serum TG levels than those with 25(OH)D ≥ 30 ng/mL. However, our data did not show a significant correlation between vitamin D levels and TC, HDL-C, or LDL-C levels, suggesting that there are regional or racial differences in the impact of vitamin D on lipid profiles because vitamin D levels are strongly related to location, life patterns, and skin pigmentation. Although several biochemical parameters were presented differently between the vitamin D deficient and sufficient groups, we found that only age, sex, and TG levels were independent risk factors for vitamin D deficiency through logistic regression analysis. Additionally, we showed that sex difference modulates the inverse association between serum TG and 25(OH)D levels. Generally, women have a higher risk of vitamin D deficiency than men. However, the negative correlation between serum TG and vitamin D levels was more significant in men, and this aligns with the findings of recent studies [36,37]. Furthermore, for the first time, we demonstrated that the association between serum vitamin D and TG levels also differs with age. The positive association between vitamin D deficiency and hypertriglyceridemia was significant in those younger than 65, but not in those older than 65 years. However, subgroup analyses revealed that vitamin D deficiency is associated with hypertriglyceridemia only among men (but not women) aged between 50 and 65 years. A randomized controlled trial showed that supplementation with vitamin D (200 IU/day for 16 weeks) did not change serum lipid levels in women aged 24.7 years [38]. Accordingly, middle-aged men with hypertriglyceridemia may be associated with a high possibility of vitamin D deficiency.

Eating habits have a great impact on TG metabolism. Higher intake of simple sugars was found to contribute to increased plasma concentrations of TG [39]. A recent analysis of 191,558 participants revealed that higher fish consumption was associated with lower TG levels [40]. Replacing simple carbohydrates with complex carbohydrates and taking omega-3 fish oil are both effective treatments for hypertriglyceridemia [41]. In addition, higher fish consumption has been demonstrated to increase serum 25(OH)D levels significantly among 402 healthy people [42]. Therefore, vitamin D may mediate the TG lowering effect of fish oil, at least in part. Rats with high-carbohydrate-diet-induced obesity exhibited higher TC, TG, and LDL-C as well as lower HDL-C levels than control rats. All of these lipid alterations were attenuated by vitamin D supplementation [43]. Vitamin D significantly reduced plasma TG and LDL-C levels in mice with high-fat diets and streptozotocin-induced diabetes mellitus [44]. Furthermore, vitamin D alleviated the increases in plasma TC, TG, and LDL-C levels and the decrease in HDL-C levels caused by ovariectomy in female rats [45]. Treatment with calcitriol, the bioactive vitamin D metabolite, decreased plasma concentrations of TG and LDL-C and increased HDL-C levels in hypercholesterolemic male rabbits [46]. In addition, sterol regulatory element-binding proteins (SREBPs) are transcription factors that modulate the lipid metabolism by upregulating the expression of lipogenic genes. Finally, 25(OH)D has been demonstrated to impair SREBP activation by inducing its proteolytic degradation [47]. These findings suggest the therapeutic potential of vitamin D for hyperlipidemia.

This study has several limitations. First, this is a cross-sectional study; thus, causality cannot be proven. Second, this study was conducted using data from patients at a single center, and the generalizability of the results to the entire Taiwanese population is limited given that the sample sizes within our subgroups were small. Third, we were unable to obtain data on many confounding factors that may influence serum vitamin D and TG levels, including medical history, smoking, dietary characteristics, sun exposure, physical activity levels, and work environment. More work is required to clarify whether the correction of vitamin D deficiency can yield clinically meaningful changes in lipid concentrations and, thereby, further reduce the risk of cardiovascular diseases.

## 5. Conclusions

Adults younger than 65 years old, women, and middle-aged men with high serum TG levels might have a higher risk of vitamin D deficiency.

## Figures and Tables

**Table 1:** Associations between vitamin D status and biomarkers in all participants.

	Vitamin D < 30 ng/mL (*n* = 665)	Vitamin D ≥ 30 ng/mL (*n* = 344)	*p* Value
Age (years)	50.02 ± 10.99	55.62 ± 11.65	<0.001
Female (%)	326 (49)	110 (32)	<0.001
BW (kg)	67.47 ± 14.21	67.39 ± 12.89	0.93
BMI (kg/m^2^)	24.48 ± 3.87	24.28 ± 3.39	0.39
WBC (10^3^/μL)	6.21 ± 1.73	6.01 ± 1.65	0.07
RBC (10^6^/μL)	4.89 ± 0.51	4.92 ± 0.52	0.37
Hb (g/dL)	14.56 ± 1.58	14.93 ± 1.33	<0.001
Hct (%)	43.14 ± 4.25	44.19 ± 3.60	<0.001
MCH (pg)	29.89 ± 2.90	30.51 ± 2.58	<0.001
MCV (fL)	88.52 ± 7.16	90.22 ± 6.52	<0.001
MCHC (g/dL)	33.71 ± 0.95	33.78 ± 0.84	0.25
Platelets (10^3^/μL)	247.1 ± 59.18	230.5 ± 54.03	<0.001
MPV (fL)	8.46 ± 0.89	8.50 ± 0.88	0.48
Fasting glucose (mg/dL)	96.85 ± 22.96	99.13 ± 22.52	0.13
HbA1c (%)	5.63 ± 0.78	5.74 ± 0.88	<0.05
BUN (mg/dL)	10.49 ± 3.55	11.37 ± 3.55	<0.001
Creatinine (mg/dL)	0.80 ± 0.24	0.85 ± 0.18	<0.005
Uric acid (mg/dL)	5.80 ± 1.42	5.95 ± 1.38	0.11
AST (U/L)	20.70 ± 8.48	22.26 ± 8.91	<0.01
ALT (U/L)	23.31 ± 16.77	24.01 ± 16.97	0.53
r-GT (U/L)	25.55 ± 26.28	27.14 ± 24.90	0.35
Alk-p (U/L)	58.73 ± 18.61	57.38 ± 15.31	0.22
Total bilirubin (mg/dL)	1.03 ± 0.45	1.07 ± 0.42	0.18
Total protein (g/dL)	7.15 ± 0.39	7.09 ± 0.40	<0.05
Albumin (g/dL)	4.46 ± 0.25	4.44 ± 0.25	0.16
Globulin (g/dL)	2.69 ± 0.34	2.65 ± 0.36	0.10
TG (mg/dL)	133.2 ± 76.04	119.9 ± 64.28	<0.005
Total cholesterol (mg/dL)	192.8 ± 39.05	193.1 ± 38.21	0.91
HDL-C (mg/dL)	51.26 ± 14.17	52.38 ± 14.60	0.24
LDL-C (mg/dL)	124.4 ± 34.06	125.4 ± 34.38	0.66
TSH (μIU/mL)	2.36 ± 3.50	2.03 ± 1.42	<0.05
T3 (ng/dL)	101.9 ± 18.76	102.7 ± 17.36	0.50
Free T4 (ng/dL)	0.94 ± 0.23	0.92 ± 0.13	0.19

BW: body weight; BMI: body mass index; WBC: white blood cell count; RBC: red blood cell count; Hb: hemoglobin; Hct: hematocrit; MCH: mean corpuscular hemoglobin; MCV: mean corpuscular volume; MPV: mean platelet volume; HbA1c: glycated hemoglobin; BUN: blood urea nitrogen; AST: aspartate transaminase; ALT: alanine transaminase; γ-GT: γ-glutamyl transpeptidase; Alk-p: alkaline phosphatase; TG: triglyceride; HDL-C: high-density lipoprotein cholesterol; LDL-C: low-density lipoprotein cholesterol; TSH: thyroid-stimulating hormone; T3: triiodothyronine; Free T4: free thyroxine.

**Table 2:** Associations between vitamin D status and biomarkers in men and women.

	Men			Women		
	Vitamin D < 30 (*n* = 339)	Vitamin D ≥ 30 (*n* = 234)	*p* Value	Vitamin D < 30 (*n* = 326)	Vitamin D ≥ 30 (*n* = 110)	*p* Value
Age (years)	50.30 ± 10.95	55.07 ± 11.91	<0.001	49.72 ± 11.04	56.77 ± 11.06	<0.001
BW (kg)	76.21 ± 12.33	72.80 ± 11.23	<0.001	58.38 ± 9.59	55.90 ± 7.58	<0.01
BMI (kg/m^2^)	25.86 ± 3.64	25.18 ± 3.29	<0.05	23.05 ± 3.57	22.36 ± 2.76	<0.05
WBC (10^3^/μL)	6.52 ± 1.70	6.21 ± 1.62	<0.05	5.88 ± 1.70	5.58 ± 1.65	0.10
RBC (10^6^/μL)	5.12 ± 0.50	5.09 ± 0.48	0.38	4.64 ± 0.40	4.56 ± 0.40	0.06
Hb (g/dL)	15.42 ± 1.30	15.41 ± 1.22	0.95	13.66 ± 1.33	13.91 ± 0.91	<0.05
Hct (%)	45.52 ± 3.56	45.54 ± 3.18	0.93	40.66 ± 3.42	41.30 ± 2.63	<0.05
MCH (pg)	30.22 ± 2.64	30.46 ± 2.86	0.29	29.55 ± 3.11	30.61 ± 1.88	<0.001
MCV (fL)	89.15 ± 6.67	89.95 ± 7.16	0.17	87.87 ± 7.59	90.81 ± 4.86	<0.001
MCHC (g/dL)	33.86 ± 0.86	33.82 ± 0.90	0.63	33.56 ± 1.02	33.69 ± 0.69	0.13
Platelets (10^3^/μL)	236.5 ± 60.34	224.0 ± 48.86	<0.01	258.0 ± 55.94	244.2 ± 61.98	<0.05
MPV (fL)	8.45 ± 0.87	8.50 ± 0.88	0.52	8.47 ± 0.91	8.50 ± 0.89	0.71
Fasting glucose (mg/dL)	100.8 ± 24.49	100.7 ± 23.35	0.97	92.76 ± 20.52	95.78 ± 20.36	0.18
HbA1c (%)	5.74 ± 0.91	5.81 ± 0.97	0.42	5.51 ± 0.61	5.62 ± 0.65	0.14
BUN (mg/dL)	11.54 ± 3.68	11.95 ± 3.44	0.18	9.40 ± 3.05	10.14 ± 3.49	<0.05
Creatinine (mg/dL)	0.95 ± 0.25	0.93 ± 0.14	0.35	0.65 ± 0.09	0.67 ± 0.10	0.14
Uric acid (mg/dL)	6.57 ± 1.28	6.42 ± 1.27	0.19	5.01 ± 1.09	4.94 ± 1.03	0.55
AST (U/L)	22.18 ± 9.68	22.83 ± 9.40	0.42	19.15 ± 6.70	21.04 ± 7.65	<0.05
ALT (U/L)	28.41 ± 19.22	25.92 ± 17.73	0.11	18.02 ± 11.63	19.95 ± 14.48	0.20
r-GT (U/L)	31.75 ± 32.72	30.79 ± 28.15	0.70	19.11 ± 14.67	19.32 ± 12.77	0.89
Alk-p (U/L)	60.02 ± 19.54	57.00 ± 14.38	<0.05	57.39 ± 17.52	58.20 ± 17.15	0.67
Total bilirubin (mg/dL)	1.13 ± 0.49	1.13 ± 0.43	0.99	0.92 ± 0.37	0.94 ± 0.36	0.75
Total protein (g/dL)	7.12 ± 0.38	7.08 ± 0.38	0.20	7.19 ± 0.40	7.12 ± 0.43	0.12
Albumin (g/dL)	4.51 ± 0.26	4.46 ± 0.26	0.06	4.41 ± 0.24	4.38 ± 0.22	0.23
Globulin (g/dL)	2.61 ± 0.33	2.61 ± 0.35	0.95	2.77 ± 0.34	2.73 ± 0.36	0.29
TG (mg/dL)	152.1 ± 83.64	128.8 ± 70.04	<0.001	113.6 ± 61.47	101.0 ± 44.50	<0.05
Total cholesterol (mg/dL)	187.6 ± 36.89	186.8 ± 38.65	0.78	198.2 ± 40.53	206.6 ± 33.63	<0.05
HDL-C (mg/dL)	44.16 ± 9.62	47.48 ± 11.46	<0.001	58.65 ± 14.38	62.80 ± 15.14	<0.05
LDL-C (mg/dL)	124.8 ± 32.88	123.8 ± 36.08	0.72	124.1 ± 35.29	128.9 ± 30.29	0.19
TSH (μIU/mL)	2.08 ± 1.40	2.01 ± 1.48	0.57	2.64 ± 4.79	2.08 ± 1.30	0.06
T3 (ng/dL)	104.8 ± 18.85	103.2 ± 16.12	0.30	98.9 ± 18.23	101.5 ± 19.77	0.20
Free T4 (ng/dL)	0.96 ± 0.27	0.93 ± 0.13	0.14	0.92 ± 0.17	0.90 ± 0.11	0.34

BW: body weight; BMI: body mass index; WBC: white blood cell count; RBC: red blood cell count; Hb: hemoglobin; Hct: hematocrit; MCH: mean corpuscular hemoglobin; MCV: mean corpuscular volume; MPV: mean platelet volume; HbA1c: glycated hemoglobin; BUN: blood urea nitrogen; AST: aspartate transaminase; ALT: alanine transaminase; γ-GT: γ-glutamyl transpeptidase; Alk-p: alkaline phosphatase; TG: triglyceride; HDL-C: high-density lipoprotein cholesterol; LDL-C: low-density lipoprotein cholesterol; TSH: thyroid-stimulating hormone; T3: triiodothyronine; Free T4: free thyroxine.

**Table 3:** Associations between vitamin D status and biomarkers in individuals aged older and younger than 65 years.

	Age ≥ 65 Years			Age < 65 Years		
	Vitamin D < 30 (*n* = 60)	Vitamin D ≥ 30 (*n* = 72)	*p* Value	Vitamin D < 30 (*n* = 605)	Vitamin D ≥ 30 (*n* = 272)	*p* Value
Female (%)	28 (46.67)	23 (31.94)	0.08	298 (49.26)	87 (31.99)	<0.001
BW (kg)	63.76 ± 11.16	62.53 ± 12.12	0.54	67.84 ± 14.43	68.68 ± 12.80	0.38
BMI (kg/m^2^)	24.86 ± 3.22	23.68 ± 3.44	<0.05	24.44 ± 0.92	24.44 ± 3.37	0.98
WBC (10^3^/μL)	6.20 ± 1.65	5.66 ± 1.29	<0.05	6.21 ± 1.74	6.10 ± 1.72	0.38
RBC (10^6^/μL)	4.65 ± 0.47	4.76 ± 0.51	0.20	4.91 ± 0.51	4.96 ± 0.52	0.20
Hb (g/dL)	14.40 ± 1.32	14.62 ± 1.25	0.32	14.57 ± 1.60	15.01 ± 1.34	<0.001
Hct (%)	42.55 ± 3.74	43.24 ± 3.37	0.26	43.19 ± 4.29	44.44 ± 3.63	<0.001
MCH (pg)	31.02 ± 2.07	30.85 ± 2.59	0.67	29.78 ± 2.94	30.42 ± 2.58	<0.005
MCV (fL)	91.60 ± 5.04	91.19 ± 6.79	0.69	88.22 ± 7.27	89.97 ± 6.43	<0.001
MCHC (g/dL)	33.85 ± 0.76	33.81 ± 0.73	0.75	33.70 ± 0.97	33.77 ± 0.87	0.28
Platelets (10^3^/μL)	221.6 ± 45.85	215.3 ± 47.09	0.43	249.6 ± 59.79	234.5 ± 55.10	<0.001
MPV (fL)	8.66 ± 0.85	8.61 ± 0.92	0.72	8.44 ± 0.89	8.47 ± 0.87	0.60
Fasting glucose (mg/dL)	105.8 ± 25.36	106.8 ± 28.55	0.83	95.97 ± 22.54	97.12 ± 20.22	0.45
HbA1c (%)	5.96 ± 0.86	6.14 ± 1.28	0.32	5.60 ± 0.77	5.64 ± 0.71	0.45
BUN (mg/dL)	12.56 ± 5.04	13.25 ± 3.74	0.38	10.29 ± 3.30	10.87 ± 3.34	<0.05
Creatinine (mg/dL)	0.86 ± 0.48	0.85 ± 0.19	0.94	0.80 ± 0.20	0.85 ± 0.17	<0.001
Uric acid (mg/dL)	5.92 ± 1.50	5.84 ± 1.35	0.74	5.79 ± 1.42	5.98 ± 1.39	0.06
AST (U/L)	22.38 ± 9.46	22.13 ± 5.74	0.86	20.53 ± 8.37	22.29 ± 9.58	<0.01
ALT (U/L)	20.86 ± 12.40	20.54 ± 7.83	0.86	23.55 ± 17.13	24.93 ± 18.56	0.28
r-GT (U/L)	27.65 ± 40.88	25.69 ± 20.97	0.73	25.34 ± 24.40	27.53 ± 25.86	0.23
Alk-p (U/L)	63.01 ± 17.45	61.37 ± 15.82	0.57	58.30 ± 18.69	56.33 ± 15.02	0.09
Total bilirubin (mg/dL)	0.96 ± 0.31	1.03 ± 0.44	0.26	1.04 ± 0.46	1.08 ± 0.42	0.21
Total protein (g/dL)	7.08 ± 0.40	7.05 ± 0.46	0.77	7.16 ± 0.39	7.10 ± 0.30	<0.05
Albumin (g/dL)	4.33 ± 0.25	4.31 ± 0.25	0.54	4.47 ± 0.25	4.47 ± 0.23	0.92
Globulin (g/dL)	2.74 ± 0.38	2.74 ± 0.42	0.94	2.68 ± 0.34	2.62 ± 0.34	<0.05
TG (mg/dL)	134.4 ± 69.92	114.2 ± 65.68	0.08	133.1 ± 76.68	121.5 ± 63.94	<0.05
Total cholesterol (mg/dL)	192.1 ± 41.31	186.5 ± 36.71	0.40	129.9 ± 38.85	194.8 ± 38.47	0.48
HDL-C (mg/dL)	51.03 ± 13.09	50.91 ± 13.40	0.96	51.29 ± 14.28	52.77 ± 14.90	0.16
LDL-C (mg/dL)	121.9 ± 40.36	120.5 ± 34.92	0.83	124.7 ± 33.33	126.7 ± 34.18	0.41
TSH (μIU/mL)	3.94 ± 10.61	2.29 ± 2.18	0.24	2.20 ± 1.49	1.96 ± 1.13	<0.05
T3 (ng/dL)	98.72 ± 20.26	100.60 ± 20.44	0.59	102.2 ± 18.60	103.2 ± 16.44	0.40
Free T4 (ng/dL)	0.90 ± 0.16	0.91 ± 0.13	0.71	0.94 ± 0.23	0.92 ± 0.13	0.21

BW: body weight; BMI: body mass index; WBC: white blood cell count; RBC: red blood cell count; Hb: hemoglobin; Hct: hematocrit; MCH: mean corpuscular hemoglobin; MCV: mean corpuscular volume; MPV: mean platelet volume; HbA1c: glycated hemoglobin; BUN: blood urea nitrogen; AST: aspartate transaminase; ALT: alanine transaminase; γ-GT: γ-glutamyl transpeptidase; Alk-p: alkaline phosphatase; TG: triglyceride; HDL-C: high-density lipoprotein cholesterol; LDL-C: low-density lipoprotein cholesterol; TSH: thyroid-stimulating hormone; T3: triiodothyronine; Free T4: free thyroxine.

**Table 4:** Associations between vitamin D status and biomarkers in men and women younger than 50 years.

	Men Younger than 50 Years			Women Younger than 50 Years		
	Vitamin D < 30 (*n* = 168)	Vitamin D ≥ 30 (*n* = 82)	*p* Value	Vitamin D < 30 (*n* = 186)	Vitamin D ≥ 30 (*n* = 27)	*p* Value
Age (years)	41.58 ± 7.11	41.61 ± 6.08	0.98	41.97 ± 6.64	41.33 ± 6.27	0.64
BW (kg)	78.49 ± 13.39	76.52 ± 12.97	0.27	59.15 ± 9.99	55.77 ± 4.65	0.08
BMI (kg/m^2^)	25.88 ± 4.09	25.56 ± 3.97	0.57	22.81 ± 3.57	21.57 ± 2.04	0.08
WBC (10^3^/μL)	6.67 ± 1.80	6.41 ± 1.72	0.28	5.88 ± 1.80	5.60 ± 1.61	0.20
RBC (10^6^/μL)	5.22 ± 0.50	5.19 ± 0.49	0.61	4.66 ± 0.43	4.46 ± 0.34	<0.05
Hb (g/dL)	15.52 ± 1.31	15.65 ± 0.99	0.42	13.51 ± 1.46	13.72 ± 0.96	0.48
Hct (%)	45.88 ± 3.53	46.21 ± 2.59	0.44	40.35 ± 3.77	40.59 ± 2.96	0.75
MCH (pg)	29.87 ± 2.77	30.34 ± 2.53	0.19	29.17 ± 3.41	30.81 ± 1.31	<0.05
MCV (fL)	88.23 ± 6.95	89.55 ± 6.59	0.15	87.01 ± 8.32	91.18 ± 3.48	<0.05
MCHC (g/dL)	33.81 ± 0.91	33.86 ± 0.75	0.69	33.45 ± 1.08	33.80 ± 0.61	0.10
Platelets (10^3^/μL)	236.5 ± 60.34	224.0 ± 48.86	0.16	268.2 ± 58.24	268.4 ± 85.49	0.99
MPV (fL)	8.44 ± 0.88	8.57 ± 0.88	0.26	8.46 ± 0.95	8.26 ± 0.90	0.30
Fasting glucose (mg/dL)	96.19 ± 15.71	93.73 ± 16.00	0.25	88.44 ± 19.96	88.15 ± 8.23	0.94
HbA1c (%)	5.56 ± 0.60	5.52 ± 0.75	0.65	5.33 ± 0.53	5.26 ± 0.28	0.48
BUN (mg/dL)	10.82 ± 3.29	10.90 ± 3.11	0.85	8.29 ± 2.39	7.37 ± 1.96	0.06
Creatinine (mg/dL)	0.95 ± 0.21	0.93 ± 0.12	0.42	0.65 ± 0.08	0.68 ± 0.08	<0.05
Uric acid (mg/dL)	6.72 ± 1.26	6.55 ± 1.3	0.32	4.83 ± 0.99	4.94 ± 0.82	0.09
AST (U/L)	22.3 ± 10.71	24.37 ± 13.00	0.18	17.58 ± 6.58	16.89 ± 2.99	0.59
ALT (U/L)	29.76 ± 21.73	30.20 ± 24.74	0.89	16.1 ± 12.03	13.11 ± 3.56	0.20
r-GT (U/L)	30.93 ± 21.24	29.39 ± 19.97	0.58	16.45 ± 9.70	12.93 ± 4.68	0.06
Alk-p (U/L)	59.6± 15.68	55.88 ± 12.21	0.06	50.71 ± 15.01	42.07 ± 12.37	<0.01
Total bilirubin (mg/dL)	1.17 ± 0.55	1.19 ± 0.48	0.75	0.95 ± 0.40	0.96 ± 0.39	0.97
Total protein (g/dL)	7.21 ± 0.37	7.12 ± 0.36	0.06	7.18 ± 0.44	7.09 ± 0.42	0.32
Albumin (g/dL)	4.58 ± 0.25	4.56 ± 0.24	0.50	4.41 ± 0.25	4.42 ± 0.24	0.85
Globulin (g/dL)	2.63 ± 0.35	2.56 ± 0.34	0.14	2.77 ± 0.35	2.67 ± 0.36	0.17
TG (mg/dL)	151.1 ± 80.28	134.2 ± 76.94	0.11	106.7 ± 58.31	85.07 ± 26.82	0.06
Total cholesterol (mg/dL)	189.6 ± 33.05	192.2 ± 39.57	0.59	188.7 ± 36.63	198.1 ± 37.69	0.21
HDL-C (mg/dL)	43.37 ± 8.89	49.2 ± 13.15	<0.001	57.76 ± 13.45	67.74 ± 14.96	<0.001
LDL-C (mg/dL)	129.0 ± 29.98	128.1 ± 36.74	0.85	117.7 ± 33.03	116.0 ± 32.25	0.80
TSH (μIU/mL)	2.00 ± 1.13	1.92 ± 1.15	0.60	2.40 ±1.84	1.87 ± 0.98	0.14
T3 (ng/dL)	107.4 ± 16.32	104.5 ± 15.22	0.18	99.41 ± 18.75	98.78 ± 21.03	0.87
Free T4 (ng/dL)	0.95 ± 0.13	0.94 ± 0.13	0.51	0.93 ± 0.19	0.89 ± 0.11	0.29

BW: body weight; BMI: body mass index; WBC: white blood cell count; RBC: red blood cell count; Hb: hemoglobin; Hct: hematocrit; MCH: mean corpuscular hemoglobin; MCV: mean corpuscular volume; MPV: mean platelet volume; HbA1c: glycated hemoglobin; BUN: blood urea nitrogen; AST: aspartate transaminase; ALT: alanine transaminase; γ-GT: γ-glutamyl transpeptidase; Alk-p: alkaline phosphatase; TG: triglyceride; HDL-C: high-density lipoprotein cholesterol; LDL-C: low-density lipoprotein cholesterol; TSH: thyroid-stimulating hormone; T3: triiodothyronine; Free T4: free thyroxine.

**Table 5:** Associations between vitamin D status and biomarkers in men and women aged between 50 and 65 years.

	Men Aged between 50 and 65 Years			Women Aged between 50 and 65 Years		
	Vitamin D < 30 (*n* = 135)	Vitamin D ≥ 30 (*n* = 100)	*p* Value	Vitamin D < 30 (*n* = 105)	Vitamin D ≥ 30 (*n* = 56)	*p* Value
Age (years)	56.55 ± 4.14	57.97 ± 4.01	<0.01	57.19 ± 3.98	58.05 ± 4.23	0.20
BW (kg)	74.95 ± 10.53	72.97 ± 9.31	0.13	57.36 ± 9.18	57.29 ± 7.63	0.95
BMI (kg/m^2^)	25.96 ± 3.17	25.29 ± 2.8	0.09	23.02 ± 3.50	22.94 ± 2.89	0.87
WBC (10^3^/μL)	6.36 ± 1.61	6.30 ± 1.69	0.74	5.55 ± 1.48	5.59 ± 1.84	0.88
RBC (10^6^/μL)	5.11 ± 0.47	5.08 ± 0.45	0.67	4.66 ± 0.32	4.67 ± 0.46	0.85
Hb (g/dL)	15.40 ± 1.28	15.41 ± 1.32	0.92	13.90 ± 1.18	14.04 ± 0.92	0.45
Hct (%)	45.44 ± 3.48	45.55 ± 3.36	0.81	41.15 ± 2.96	41.75 ± 2.59	0.21
MCH (pg)	30.24 ± 2.57	30.50 ± 3.14	0.50	29.91 ± 2.70	30.22 ± 2.23	0.47
MCV (fL)	89.25 ± 6.54	90.04 ± 7.54	0.39	88.54 ± 6.59	89.79 ± 5.63	0.23
MCHC (g/dL)	33.85 ± 0.83	33.81 ± 1.06	0.70	33.74 ± 0.97	33.63 ± 0.78	0.47
Platelets (10^3^/μL)	235.9 ± 72.80	223.4 ± 50.39	0.14	245.7 ± 53.23	240.8 ± 51.92	0.58
MPV (fL)	8.43 ± 0.88	8.47 ± 0.85	0.72	8.43 ± 0.85	8.44 ± 0.87	0.95
Fasting glucose (mg/dL)	105.1 ± 31.59	102.6 ± 25.36	0.52	96.04 ± 16.47	95.34 ± 17.58	0.80
HbA1c (%)	5.95 ± 1.16	5.80 ± 0.81	0.28	5.68 ± 0.52	5.64 ± 0.55	0.60
BUN (mg/dL)	12.00 ± 3.10	12.09 ± 3.17	0.83	10.62 ± 3.30	9.91 ± 2.84	0.18
Creatinine (mg/dL)	0.94 ± 0.15	0.95 ± 0.15	0.71	0.67 ± 0.11	0.65 ± 0.10	0.46
Uric acid (mg/dL)	6.44 ± 1.27	6.39 ± 1.24	0.76	5.21 ± 1.11	5.07 ± 1.04	0.47
AST (U/L)	21.87 ± 7.62	21.80 ± 6.82	0.94	21.04 ± 6.77	22.45 ± 9.40	0.28
ALT (U/L)	27.91 ± 16.68	24.86 ± 13.17	0.13	21.31 ± 11.67	23.08 ± 18.65	0.45
r-GT (U/L)	29.26 ± 17.68	33.53 ± 34.96	0.22	22.90 ± 18.12	20.23 ± 10.72	0.32
Alk-p (U/L)	60.99 ± 24.11	56.98 ± 15.43	0.15	66.22 ± 17.00	61.50 ± 15.90	0.09
Total bilirubin (mg/dL)	1.10 ± 0.46	1.11 ± 0.41	0.81	0.91 ± 0.36	0.95 ± 0.33	0.51
Total protein (g/dL)	7.07 ± 0.38	7.04 ± 0.36	0.50	7.18 ± 0.38	7.23 ± 0.41	0.51
Albumin (g/dL)	4.47 ± 0.25	4.47 ± 0.23	0.83	4.44 ± 0.24	4.42 ± 0.24	0.64
Globulin (g/dL)	2.60 ± 0.32	2.57 ± 0.31	0.54	2.75 ± 0.31	2.81 ± 0.34	0.25
TG (mg/dL)	161.6 ± 92.25	131.0 ± 65.82	<0.01	117.1 ± 58.62	103.5 ± 40.49	0.12
Total cholesterol (mg/dL)	185.9 ± 41.41	186.5 ± 37.74	0.91	211.5 ± 41.89	215.0 ± 31.42	0.59
HDL-C (mg/dL)	44.11 ± 9.56	46.38 ± 9.30	0.07	60.56 ± 15.21	62.91 ± 17.49	0.38
LDL-C (mg/dL)	120.2 ± 34.84	125.0 ± 35.79	0.31	133.8 ± 34.51	135.7 ± 27.04	0.72
TSH (μIU/mL)	2.10 ± 1.48	1.96 ± 1.13	0.44	2.41 ±1.36	2.00 ± 1.13	0.06
T3 (ng/dL)	103.3 ± 20.93	103.9 ± 15.73	0.80	97.89 ± 16.79	100.6 ± 16.45	0.33
Free T4 (ng/dL)	0.97 ± 0.40	0.94 ± 0.14	0.42	0.92 ± 0.14	0.92 ± 0.12	0.76

BW: body weight; BMI: body mass index; WBC: white blood cell count; RBC: red blood cell count; Hb: hemoglobin; Hct: hematocrit; MCH: mean corpuscular hemoglobin; MCV: mean corpuscular volume; MPV: mean platelet volume; HbA1c: glycated hemoglobin; BUN: blood urea nitrogen; AST: aspartate transaminase; ALT: alanine transaminase; γ-GT: γ-glutamyl transpeptidase; Alk-p: alkaline phosphatase; TG: triglyceride; HDL-C: high-density lipoprotein cholesterol; LDL-C: low-density lipoprotein cholesterol; TSH: thyroid-stimulating hormone; T3: triiodothyronine; Free T4: free thyroxine.

## Data Availability

The data presented in this study are available on request from the corresponding author. The data are not publicly available due to their containing information that could compromise the privacy of research participants.
